# Autoimmune Thyroid Disease in Specific Genetic Syndromes in Childhood and Adolescence

**DOI:** 10.3389/fendo.2020.00543

**Published:** 2020-08-19

**Authors:** Eleni Magdalini Kyritsi, Christina Kanaka-Gantenbein

**Affiliations:** Division of Endocrinology, Diabetes and Metabolism, First Department of Pediatrics, Medical School, Aghia Sophia Children's Hospital, National and Kapodistrian University of Athens, Athens, Greece

**Keywords:** autoimmune thyroid disease, Hashimoto, genetic syndromes, Turner syndrome, Down syndrome, Klinefelter syndrome

## Abstract

Autoimmune thyroid disease (ATD) is the most frequent cause of acquired thyroid dysfunction, most commonly presenting either as Hashimoto's thyroiditis or Graves' Disease. Hashimoto's thyroiditis is characterized by the presence of thyroid-specific autoantibodies, more commonly anti-thyroperoxidase antibodies in the serum and the typical inhomogeneous echostructure of the thyroid on a thyroid ultrasound examination. Hashimoto's thyroiditis can for a long time be accompanied by normal thyroid function and hypothyroidism can only progressively be established. Graves' disease is much less frequent in childhood and adolescence and presents with overt hyperthyroidism. After the onset of puberty, ATD affects females with a higher incidence than males, while during the prepubertal period there is not such a clear preponderance of affected females. ATD can occur either isolated or in the context of other autoimmune disorders, such as type 1 Diabetes mellitus (T1D), celiac disease, alopecia areata, vitiligo, etc. Especially at the pediatric age, a higher incidence of ATD is also observed in the context of specific genetic syndromes, such as trisomy 21 (Down syndrome), Klinefelter syndrome, Turner syndrome, or 22q11.2 deletion syndrome. Nevertheless, although thyroid dysfunction may also be observed in other genetic syndromes, such as Prader-Willi or Williams syndrome, the thyroid dysfunction in these syndromes is not the result of thyroid autoimmunity. Interestingly, there is emerging evidence supporting a possible link between autoimmunity and RASopathies. In this review article the incidence, as well as the clinical manifestation and accompanied pathologies of ATD in specific genetic syndromes will be presented and regular follow-up for the early identification of the disorder will be proposed.

## Introduction

### Autoimmune Thyroid Disease in Children and Adolescents

Autoimmune thyroid disease (ATD) is the most common thyroidopathy in children and adolescents. It comprises two main entities, Hashimoto's Thyroiditis (HT) and Graves' Disease (GD) and a wide spectrum of clinical presentations, ranging from Hashimoto's overt or subclinical hypothyroidism, euthyroidism, to Graves' subclinical or overt hyperthyroidism. Conversion of hyperthyroidism to hypothyroidism and vice versa may also occur, implying that these two disorders may represent opposite sides of the same coin. ATD results from the complex interplay between genetic, environmental and endogenous factors leading to loss of self-tolerance to thyroid antigens. Both cell-mediated and humoral immune responses are implicated in the pathogenesis of ATD. Activation of T- and B cell pathways drives the infiltration of the thyroid by autoreactive lymphocytes and the production of antibodies against three main thyroid antigens, thyroid peroxidase (TPO), thyroglobulin (TG), and thyroid-stimulating hormone receptor (TSHR). While in HT the autoimmune processes lead to apoptosis and destruction of the thyroid follicles, and subsequently hypothyroidism, in GD the immune-mediated activation of TSHR-reactive B cells results in the production of stimulating TSHR antibodies which in turn induce thyroid cell proliferation and function, manifested as hyperthyroidism (1, 2). Around 70% of the risk for developing ATD can be ascribed to genetic factors. It is noteworthy, that thyroid autoantibodies can be found in ~50% of first-degree relatives of patients with ATD (3). Some of the genes that have been shown to confer susceptibility to ATD include (1) immune modulating genes: Human leukocyte antigen/HLA-DR, cytotoxic T-lymphocyte-associated protein 4/CTLA-4, cluster of differentiation 40/CD40, protein tyrosine phosphatase, non-receptor type 22 (lymphoid) PTPN22 and (2) thyroid specific genes: TG and TSHR (1, 4). Moreover, hormones, mainly estrogens, as also observed during pregnancy, stress, smoking, iodine, infection, drugs (lithium, amiodarone, interferon-alpha), radiation and exposure to environmental toxins may also contribute to the occurrence of ATD in genetically susceptible individuals (1, 5).

The frequency of HT in the pediatric age ranges between 0.3 and 9.6% (around 3%), occurring rarely before the age of 3 years and reaching a peak in early to mid-puberty. There is a strong female preponderance with a female-to-male (F/M) ratio varying across studies between 2:1 and 9.7:1 (6–8). This female predominance is less pronounced in prepubertal children (F/M ratio: 1.6), suggesting the influence of sex hormones in the development of ATD (9). Diagnosis is established by detecting positive serum TPO and/or TG autoantibodies along with a heterogeneous echotexture and diffuse or irregular hypoechogenicity of the thyroid parenchyma on ultrasound scan (1, 6).

Patients with HT are commonly asymptomatic, may present with goiter or be fortuitously diagnosed during investigations for other reasons, such as growth retardation. Moreover, HT can be detected in regular follow-up studies of children that may have an inherent increased risk for ATD, for instance subjects affected by extrathyroidal autoimmune disorders or certain chromosomal abnormalities. At the time of diagnosis, 52.1% of patients are euthyroid, 22.2% have overt and 19.2% subclinical hypothyroidism (SH). The remaining 6.5% of cases present with hyperthyroidism either overt (3.5%) or subclinical (3%) (10). With regards to the natural history of the disease, Aversa et al. evaluated the long-term evolution of thyroid status in 234 children with HT and either euthyroidism or SH at baseline. During a 5 year follow-up, a substantial proportion of patients showed a deterioration of their thyroid function as evidenced by an increase in thyroid-stimulating hormone (TSH) and a decrease in free thyroxine (FT4) levels as well as an increase in thyroid volume in the whole study population. Among patients who were initially euthyroid, 30.6% developed SH and 12.3% overt hypothyroidism. In addition, of those who had SH at baseline, 31.2% progressed to overt hypothyroidism, 3.2% developed hyperthyroidism, and 25% remained in the SH stage. It is worth mentioning, however, that among patients who were initially euthyroid 57.1% remained euthyroid at the end of the follow-up period. Moreover, spontaneous recovery of thyroid function was noted in 40.6% of cases who had SH at baseline (11).

Further data indicate that the presence of goiter and elevated TG autoantibodies at HT diagnosis may predict a deterioration of thyroid function over time (12). Moreover, in patients with HT, elevated TSH levels and TPO antibodies at diagnosis, a progressive increase in TSH during follow-up a well as the concomitant presence of celiac disease were shown to increase the risk of developing hypothyroidism after a 3 year period (7, 13).

Overall, the natural course of SH is worse in patients with underlying HT compared to idiopathic ones (14). With regards to treatment, levothyroxine replacement therapy in patients with SH is generally recommended, if TSH levels are higher than 5 IU/ml in the presence of goiter or positive thyroid antibodies and in all cases where TSH levels exceed 10 IU/ml (1).

GD, the most frequent cause of hyperthyroidism, is uncommon in the pediatric age range, with a prevalence ranging between 1/10.000 in the United States to 1/100.000 in the UK and Ireland. Similarly to HT, GD occurs 4–5 times more often in females than males, but rarely under the age of 4 years and its frequency peaks during puberty. Further to typical manifestations observed in adults, pediatric GD patients may show a decline in their school performance, behavioral changes and acceleration of growth and bone maturation. Antithyroid medications are used as first line treatment, although lower long-term remission rates have been reported in children than in adults, with <30% of patients achieving lasting remission following 24 months of medical therapy, requiring either another course of antithyroid drugs or definitive treatment (either radioiodine with I-131 or thyroidectomy) (1, 15).

The association between ATD and extrathyroid autoimmune diseases has been well-documented. While arthropathies and connective tissue disorders were shown to be the most common coexisting autoimmune disorders in adults with ATD, celiac disease (CD) and type 1 diabetes mellitus (T1D) were the most prevalent ones among ATD children and adolescents. Skin diseases, namely vitiligo, were almost equally represented among adults and children (16–18). Similarly, among GD patients with a mean age of 43 years, the most common non-thyroidal autoimmune diseases (NTAD) were rheumatoid arthritis, followed by vitiligo and pernicious anemia. In contrast, vitiligo, followed by T1D and CD were the NTADs mostly seen in pediatric GD patients (16).

Interestingly, patients with certain genetic abnormalities, namely Turner syndrome (TS), Trisomy 21 or Down syndrome (DS) and 22q11.2 deletion syndrome (22q11.2DS) are prone to develop both ATDs and NTADs. In addition, association with these syndromes may influence not only the clustering of extrathyroid autoimmune disorders but also the evolution of thyroid function in patients with ATD (16, 19).

In this review, we will present the underlying mechanisms, frequency, and natural course of thyroid function disorders in specific genetic syndromes, providing in parallel recommendations for early identification, follow-up, and monitoring of thyroid disorders in affected individuals.

## Turner Syndrome (TS)

TS is a rare chromosomal disorder, affecting 1 in 2,000–2,500 live born females. It results from partial or complete loss of one of the X chromosomes and is associated with a constellation of clinical features including short stature, gonadal dysgenesis, dysmorphic features, cardiovascular and renal anomalies, sensorineural hearing loss, skeletal malformations, ophthalmological abnormalities, neurocognitive impairment of variable severity and lymphedema (20, 21). Mortality is increased by ~3-fold, especially due to cardiovascular diseases, congenital malformations, endocrine, nutritional, and metabolic diseases, resulting in a significant decrease in life expectancy (22).

Furthermore, women with TS are prone to develop autoimmune diseases, the most common being ATD (23–25). Moreover, there is a 4- to 8-fold increased risk of CD over the general population (26, 27). Other associated autoimmune conditions include T1D, inflammatory bowel diseases, alopecia areata, vitiligo, psoriasis, lichen sclerosus, juvenile idiopathic arthritis (JIA), and idiopathic thrombocytopenic purpura (ITP) (23, 25, 27). The risk of developing autoimmune diseases with a male predominance, such as T1D, Dupuytren's contracture, amyotrophic lateral sclerosis, ankylosing spondylitis and reactive arthritis is ~4-fold increased, whereas the risk for female-predominant autoimmune diseases is increased by 1- and 7-fold (25) (Table 1). The frequency of autoimmune disorders increases with age and two or more organ-specific autoantibodies or autoimmune conditions may coexist in the same patient (24, 27).

**Table 1:** (a) Prevalence of autoimmune thyroid disorders and (b) associated non-autoimmune thyroid disorders in pediatric patients with TS, DS, KS, 22q11.2DS, WS, PWS, NS, and NF1 (c) possible underlying mechanisms involved in the pathogenesis of autoimmunity and (d) extrathyroidal autoimmune disorders associated with these syndromes.

	**TS**	**DS**	**KS**	**22q11.2DS**
a) ATD (prevalence)	HT (10–42%)^*^ GD (1.7–3%)	HT (13–46%) GD (6.5‰)	HT (5.4–10%) GD (rare)	HT: (5% children, 30% age>17 years) GD (1.8%)
b) Non-autoimmune thyroid disorder	Primary hypothyroidism	CH - Incidence: 1:113–1:141 DS live births - mainly due to thyroid hypoplasia Primary hypothyroidism	Central hypothyroidism Peripheral hypothyroidism	Thyroid gland anomalies/hypoplasia (~50%) Primary hypothyroidism
c) Possible underlying mechanisms implicated in the pathogenesis of autoimmunity	- X-chromosome genes haploinsufficiency - parental X chromosome origin - excessive production of pro-inflammatory cytokines - decreased levels of anti-inflammatory cytokines hypogonadism	- thymic atrophy and diminished expansion of T and B lymphocytes - altered thymic expression of AIRE - association with MHC class II DQA 0301 allele - altered regulation of pro- and anti-inflammatory cytokines - hyperresponsiveness to IFN	- immunomodulatory role of sex hormones in the immune response - X-linked gene dosage	- absent/hypoplastic thymus, ↓AIRE expression - T-cell lymphopenia, ↓ Tregs - restricted T cell repertoires, abnormal T cell activation, B cell dysregulation, Th1/Th2 imbalance - association with HLA-DR14
d) Associated extrathyroidal autoimmune disorders	- CD: ↑risk 4- to 8-fold - T1D, IBD, alopecia areata, vitiligo, psoriasis, lichen sclerosus, JIA, ITP - ↑ risk 4-fold for AD with a male predominance (T1D, Dupuytren's contracture, amyotrophic lateral sclerosis, ankylosing spondylitis and reactive arthritis)	- In particular: alopecia and vitiligo - CD: ↑ risk >6 years of age - T1D, idiopathic arthritis - Addison disease, chronic autoimmune hepatitis, primary sclerosing cholangitis^**^	- SLE: ↑risk 14-fold - T1D - Addison's disease, multiple sclerosis, RA, Sjogren's syndrome - JIA, psoriatic arthritis, polymyositis/dermatomyositis, systemic sclerosis, mixed connective tissue disease, antiphospholipid syndrome, ankylosing spondylitis, primary biliary cirrhosis	- JIA, ITP (next most common causes after ATD) - autoimmune hemolytic anemia, autommune neutropenia, psoriasis, vitiligo, CD, pernicious anemia/atrophic gastritis, IBD, urticaria, Raynaud phenomenon, rheumatic fever with chorea, T1D
	**WS**	**PWS**	**NS**	**NF1**
a) ATD (prevalence)	Rare	Rare	HT (14.3–60%)	HT: 2.5% GD: rare
b) Non-autoimmune thyroid disorder	Thyroid hypoplasia (75%) - Other structural abnormalities: agenesis, hemiagenesis and ectopy Primary hypothyroidism CH (rare) Hyperthyroidism (rare) Central hypothyroidism (rare)	Mainly central hypothyroidism (6.8%) Primary hypothyroidism CH Ectopic thyroid gland	Primary hypothyroidism	Central hypothyroidism Primary hypothyroidism
c) Possible underlying mechanisms implicated in the pathogenesis of autoimmunity			- both under- and overactivities of disparate Ras effectors - both increased and decreased Ras activities may be implicated in lupus-like autoimmunity - linkage of a susceptibility gene for SLE to 12q24, a locus encompassing *PTPN11*, encoding SHP-2 SHP-2 inhibits NK cells activation, cytolytic activity and IFN-γ	- loss of neurofibromin resulting in decreased Fas antigen expression which may prevent apoptosis of CD4+ T cells - lymphoproliferative defects, including thymic and splenic hyperplasia, increased numbers of immature and mature T cells *in vivo*, but reduced proliferation in response to TCR and
			secretion by NK cells, may mediate inactivation of immunoregulatory receptors and functions as a regulator of NF-κB activation. Increased SHP-2 activity is involved in SLE pathogenesis, modulating T cell proliferation and downstream cytokine production	IL-2R stimulation *in vitro*, defective proliferative responses in B lymphocytes and thymocytes were shown in NF1-deficient mice
d) Associated extrathyroidal autoimmune disorders	- CD^***^	- Autoantibodies against pituitary	- Vasculitis, vitiligo, anterior uveitis, SLE, CD, antiphospholipid syndrome, and autoimmune hepatitis	- Multiple sclerosis, SLE, membranous glomerulonephritis, IgA nephropathy, mixed connective tissue disease, myasthenia gravis, ankylosing spondylitis, JIA, CD, autoimmune hemolytic anemia, bullous pemphigoid, vitiligo, alopecia areata, T1D

*TS, Turner syndrome; DS, Down syndrome; KS, Klinefelter syndrome; 22q11.2DS, chromosome 22q11.2 deletion syndrome; WS, Williams syndrome; PWS, Prader-Willi syndrome; NS, Noonan syndrome; NF1, Neurofibromatosis type 1; ATD, autoimmune thyroid disease; HT, Hashimoto's thyroiditis; GD, Graves'disease; CH, congenital hypothyroidism; AIRE, autoimmune regulator; MHC, major histocompatibility complex; IFN, interferon; ↓, decreased; Tregs, regulatory T cells; Th, T helper (cell); HLA, human leukocyte antigen; PTPN11, tyrosine-protein phosphatase non-receptor type 11; SHP-2, Src homology phosphotyrosyl phosphatase 2; NK, natural killer (cells); NF-κB, nuclear factor kappa B; CD4, cluster of differentiation 4; IL-2R, interleukin-2 receptor; TCR, T cell receptor; CD, celiac disease; ↑, increased; T1D, type 1 diabetes mellitus; IBD, inflammatory bowel disease; JIA, juvenile idiopathic arthritis; RA, rheumatoid arthritis; ITP, idiopathic thrombocytopenic purpura; AD, autoimmune disease; SLE, systemic lupus erythematosus*.

* *Livadas et al. (28), Aversa et al. (29)*.

** *Giménez-Barcons et al. (30)*.

*** *Giannotti et al. reported an increased prevalence of CD in WS subjects (31), whereas Stagi et al. found no evidence of increased autoimmunity in their patients with WS, including CD (32)*.

The pathogenetic mechanisms underlying the increased frequency of autoimmune conditions among women with TS may include X-chromosome genes haploinsufficiency, parental X chromosome origin, excessive production of pro-inflammatory cytokines, decreased levels of anti-inflammatory cytokines and hypogonadism (23, 27, 33–35) (Table 1).

Haploinsufficiency of genes on the X chromosome may result in a lack of exposure to self-antigens in the thymus and subsequently inadequate thymic deletion of autoreactive T-lymphocytes and impaired “self” antigen recognition and tolerance (23, 34–36). The X chromosome is known to contain many immune-related genes (36, 37), one of them being FOXP3 (forkhead box P3). FOXP3, a member of the forkhead family of transcription factors is an essential transcriptional regulator for the development and suppressive function of regulatory T cells (Tregs), a subset of CD4+ lymphocytes (35, 38). Of note, Zinn et al. have mapped a possible locus for autoimmune thyroid disease in TS to a critical region of X chromosome, Xp11.2-p22.1, which also contains FOXP3 gene (38, 39). Mutations in the FOXP3 gene cause a rare disorder inherited in males, known as IPEX syndrome (Immune dysregulation, polyendocrinopathy, enteropathy, X-linked) that can be also characterized by ATD (38).

Immune alterations observed in TS women include a decrease in the CD4+ to CD8+ lymphocyte ratio in the peripheral blood, lower IgG levels and percentage of CD4+ lymphocytes, as well as higher percentage of CD8+ T cells and frequencies of effector memory CD4+ T cells compared to controls (27, 33, 38, 40). Furthermore, levels of pro-inflammatory cytokines (IL6 and TGF β1) were found to be increased in women with TS, whereas those of anti-inflammatory cytokines (IL10 and TGF β2) decreased (35).

With regards to Tregs the results are inconclusive. In the study of Lee et al., the Tregs of TS patients displayed impaired ability to suppress the proliferation of autologous effector CD4+T cells compared to controls, despite their higher frequency among CD4+ T cells. The above findings could indicate that the Tregs of TS patients are intrinsically defective at inhibiting the proliferation of effector T cells, and/or that the effector T cells of TS patients are resistant to the suppressive effects of Tregs (38).

On the other hand, Gawlik et al. found that the percentage of Tregs in girls with TS and coexisting autoimmune disease was lower than in healthy controls and TS girls with no autoimmune diseases (33).

PTPN22 encodes a lymphoid-specific phosphatase which acts as a negative regulator of T cells and its polymorphisms have been linked to several autoimmune disorders. In particular, the PTPN22 C1858T polymorphism was associated with autoimmune disease risk in a Brazilian population of TS, however, these findings were not replicated in Hispanic (Mexican) TS patients (41, 42), indicating the influence of different genetic backgrounds on the phenotypic expression of this polymorphism.

It has been shown that the majority (60–80%) of 45,X women retain their maternally derived X chromosome (43–45). However, the frequency of hypothyroidism or thyroid autoimmunity did not differ according to the parental origin of the X chromosome (43, 45). On the other hand, further studies supported that the inheritance of autoimmunity in TS women was preferentially paternally transmitted, since patients were more likely to harbor autoantibodies when their fathers had autoantibodies rather than their mothers. Moreover, HLA-DR7;DQ2 and HLA-DR7;DQ9 haplotypes were associated with autoimmunity in TS patients and were more often paternally transmitted (46).

Interestingly, Bakalov et al. revealed a stepwise increase in the prevalence of HT: from 3.4% in men, to 5.8% in the general US female population, 15% in women with idiopathic 46,XX primary ovarian insufficiency (POI) and 37% in women with TS, suggesting that androgen deficiency may be implicated in the pathogenesis of HT (35). The levels of androgens are reduced in women with 46,XX spontaneous premature ovarian failure and even lower in women with TS. Given the known immunosuppressive role of androgens, higher androgen levels might have a protective effect in men, whereas low androgen levels might be linked to an increased risk of HT in women with ovarian insufficiency (35, 47–49). Indeed, Kalantaridou et al. demonstrated lower serum free testosterone concentrations in women with 46,XX spontaneous premature ovarian failure (while off estrogen therapy) compared to control women with normal ovarian function. Interestingly, free testosterone concentrations dropped even lower while these patients were on physiologic transdermal E2 therapy and despite the fact that levels of sex hormone-binding globulin did not alter significantly. A possible explanation for this finding could be that estrogen therapy might have induced a relative androgen deficiency. Given that androgens are secreted by both ovaries and adrenals and LH receptors have been identified in the zona reticularis and the deeper layer of the zona fasciculata of adrenal glands, the induction of E2 therapy might have resulted in lower LH levels and consequently a reduction in the production of testosterone from both the ovary and the adrenals (47). Moreover, the study of Gravholt et al., revealed a 25–40% reduction of circulating androstendione, testosterone, free testosterone, and dihydrotestosterone in TS women while off hormone replacement therapy, compared to age-matched normal women, whereas the level of dehydroepiandrosterone sulfate (DHEAS) was comparable between TS and control subjects. Most women with TS have no ovarian function and this might explain the difference in levels of androgens. On the other hand, the above results could point toward insufficient conversion from DHEAS into androstendione, testosterone, and dihydrotestosterone in TS, given the normal level of circulating DHEAS and thus insufficient extraadrenal and extragonadal 3b-hydroxysteroid dehydrogenase and 17bhydroxysteroid dehydrogenase activity (48).

Taken into account the data of Bakalov et al., showing a higher prevalence of HT in both TS and POI women, it seems that ovarian insufficiency *per se* may be a risk factor for HT, whereas the absence of a normal second X chromosome and the resultant haploinsufficiency for X-chromosome related gene(s) may further increase the risk for thyroid and possibly other autoimmune disorders (35).

The impact of a specific karyotype on the prevalence of thyroid autoimmunity in TS women has been also addressed with conflicting results. Increased frequency of thyroid autoimmunity has been observed among TS patients with isochromosome Xq and isolated Xp deletion (33, 50–52). Common feature of the aforementioned chromosomal abnormalities is the lack of the short arm of the X chromosome (Xp), indicating that haploinsufficiency of immune-related genes located in the Xpter-p11.2 region may predispose to the development of thyroid autoimmunity in women with TS (51). The reported prevalence of positive thyroid autoantibodies in TS women with Xq-isochromosome ranges from 15 to 83% (28). Other studies have failed to find an association between thyroid autoimmunity and specific TS karyotypes (25, 53). Of interest, an increased risk of ulcerative colitis (25) and a higher prevalence of anti-GAD-65 have been documented in TS patients with isochromosome Xq karyotype (24).

The frequency of HT is far higher among TS women than in the general population (29, 54), with the prevalence increasing with age, from 15.4% in patients younger than 10 years to 30.7% in the third decade of life (55) (Table 1). Up to ~50% develop thyroid autoantibodies (50, 56). The frequency of thyroid autoimmunity rises steeply after the age of 13 years (53), but thyroid autoantibodies may appear as early as 5.5 years of age. An estimated 15–40% of patients suffer from hypothyroidism (24, 28, 50, 52, 53, 55), with its annual incidence being 3.2% (57) (Table 1). The risk of developing subclinical hypothyroidism has been shown to be highest between the ages of 12 and 14 years (53). With regards to its natural course and hormonal pattern HT in TS is characterized by the following features (1) a milder biochemical picture at diagnosis, given the higher frequency of euthyroidism and the lower TSH levels compared to non-TS subjects. These findings may be related either to the increased physicians' awareness on TS-associated thyroid dysfunction, resulting in an earlier detection of cases or to a milder autoimmune pattern as indicated by the lower TPOAb serum concentrations among TS patients (2) a progressive deterioration of thyroid function over time toward either hypo- or hyperthyroidism. Notably, Aversa et al. reported that the majority (67.7%) of TS patients presenting initially with SH evolved to overt hypothyroidism after a median time interval of 4.9 years (3) a lower frequency of positive family history of thyroid disease (4) more frequent conversion to GD (58).

According to the latest clinical practice guidelines for the care of girls and women with TS, screening for hypothyroidism is recommended at diagnosis and then in annual intervals, with assessment of (free) T4 and TSH levels starting from early childhood throughout life. Thyroid antibodies testing is usually recommended in the presence of elevated TSH levels and/or goiter (53, 59). Taken into consideration that hypothyroidism in TS may occur even before the age of 2 years (53) and could impair growth during childhood and puberty and aggravate the already adverse cardiometabolic profile of these patients, careful endocrinological surveillance is warranted across the lifespan to early detect and appropriately treat thyroid dysfunction and other comorbidities and thus optimize medical management of this population (23, 60) (Table 2).

**Table 2:** Summary of recommendations on diagnosis and management of thyroid dysfunction in pediatric patients with specific genetic syndromes.

TS	Screen for hypothyroidism as soon as the diagnosis is established and then in annual intervals, with assessment of (free) T4 and TSH levels starting from early childhood throughout life Thyroid autoantibodies testing is recommended at first detection of thyroid dysfunction and/or goiter LT4 substitution therapy should be initiated in patients with OH, and is recommended for those with SH and TSH levels >10 mIU/l and/or positive thyroid antibodies and/or suggestive signs and symptoms (such as goiter, growth deceleration etc.) Treatment modalities for GD include antithyroid medication, radioactive iodine therapy, or thyroidectomy
DS	Screen for CH at birth and repeat TSH measurements at 6 and 12 months and annually thereafter. Additional assessment of TSH and FT4 at both 6–8 weeks and 4 months of age may be advised If TSH levels are mildly elevated, consider reassessment of FT4 and TSH levels in a 2–3 month time, along with measurement of thyroid autoantibodies Obtain thyroid antibodies at least once every 2 years, from age 1 year and throughout life In the presence of positive antithyroid antibodies TSH and FT4 levels should be monitored regularly Treatment with LT4 is advisable in patients with SH and (a) TSH levels >10 μU/ml (b) TSH levels <10 μU/ml in the presence of goiter and/or positive thyroid antibodies. Special considerations should be given in infants and children below the age of 5 years LT4 therapy should be initiated in all cases that progress to overt hypothyroidism or become symptomatic A trial off therapy and re-evaluation of thyroid function tests should be offered in those patients who do not require increase in their LT4 dose following treatment initiation or displayed no rise of TSH >10 μU/ml while on treatment. Regular auxological and clinical assessment and life-long monitoring of the thyroid function are recommended for all patients Patients with GD can be offered antithyroid medication or radioactive iodine. Of note, surgery may not be an optimal choice for DS patients with the GD, as their short necks, craniofacial anomalies and associated airway obstruction may predispose to increased anesthetic and surgical risks
KS	Measurement of TSH and FT4 levels at diagnosis and annually thereafter is advised. Screening for thyroid antibodies should be considered in case of TSH elevation and/or presence of goiter or periodically in the absence of suggestive clinical or biochemical signs
22q11.2DS	Obtain TSH, FT4, total T3, and anti-TPO antibodies at diagnosis and annually thereafter Appropriate treatment should be initiated in case of overt thyroid dysfunction In case of subclinical thyroid disease, TFTs can be repeated in a 4–6 months' time interval If subclinical disease persists (and in case of overt thyroid disease), perform a thyroid ultrasound in order to rule out structural anomalies of the gland Medical treatment for subclinical disease should be offered if TSH levels remain abnormal despite normal FT4 levels in the presence of suggestive signs/symptoms and/or positive TPO antibodies and/or goiter or thyroid hypoplasia. Alternatively, TFTs can be rechecked after another 4–6 months' time interval
WS	TFTs should be performed at diagnosis, annually for the first 3 years and at 2 years' intervals thereafter Ultrasonographic assessment of thyroid morphology would be advised as soon as the diagnosis is established and regularly until adulthood In patients younger than 6 years, or in those with thyroid hypoplasia, close monitoring of the thyroid function in shorter intervals, i.e., at 3–6 months from baseline, and then yearly, should be considered LT4 treatment should be started in patients with overt hypothyroidism Ongoing monitoring may be considered for cases with SH. LT4 replacement therapy is advisable, especially in children younger than 3 years, if TSH levels are persistently elevated, in the presence of thyroid hypoplasia and/or suggestive signs and symptoms
PWS	Obtain TSH and FT4 levels within the first 3 months of life, regardless of the newborn screening result, given that TSH-based screening strategies cannot detect central hypothyroidism TSH and FT4 should be measured on an annual basis, with consideration of more frequent testing if the patient receives treatment with rGH Appropriate LT4 therapy should be initiated, if indicated. Taken into account the higher frequency of hypothyroidism during the first 2 years of life, a critical period for both growth and development, special consideration should be given in infancy and early childhood, to ensure adequate thyroid hormone levels
NS and other RASopathies	NS TFTs including measurement of thyroid antibodies should be performed in children with signs or symptoms of hypothyroidism and every 3-−5 years in older children and adults Thyroid disorders should be managed as in general population CFC Obtain FT4 and TSH levels at diagnosis During follow-up thyroid function should be checked in children with growth failure. Ongoing monitoring of the thyroid function can be decided by the endocrinologist NF1 It would be advisable to apply the same recommendations on thyroid function monitoring as for NS patients

*TS, Turner syndrome; DS, Down syndrome; KS, Klinefelter syndrome; 22q11.2DS, chromosome 22q11.2 deletion syndrome; WS, Williams syndrome; PWS, Prader-Willi syndrome; NS, Noonan syndrome; CFC, Cardio-facio-cutaneous syndrome; NF1, Neurofibromatosis type 1; T4, thyroxine; FT4, free thyroxine; TSH, thyroid stimulating hormone LT4, levothyroxine; OH, overt hypothyroidism; SH, subclinical hypothyroidism; CH, congenital hypothyrodism; GD, Graves'disease; T3, triiodothyronine; anti-TPO, anti-thyroid peroxidase (TPO); TFTs, thyroid function tests; rGH, recombinant growth hormone*.

GD, although relatively rare, occurs more frequently in TS girls compared to the pediatric general population with an estimated prevalence of 1.7–3 vs. 1.07%, respectively (28, 29, 61) (Table 1). The prevalence rates may vary across studies depending on the patient population ethnicity, age at presentation or the length of follow-up. TS patients with GD present more frequently extrathyroidal associated autoimmune disorders, are older at disease diagnosis and have lower FT4 levels than non-TS subjects. The above findings are in line with previous observations showing an increasing prevalence of ATD with age as well as a higher susceptibility of TS girls to autoimmune diseases. Moreover, regular clinical and laboratory assessment of those patients may enable the detection of thyroid dysfunction at an earlier stage (29, 61). Of interest in patients with TS or Down Syndrome (DS), GD may evolve from antecedent HT more commonly (25.7%) and later during the course of the disease compared to GD patients with no TS/DS (62). The clinical course of the disease does not seem to differ between patients with and without TS, given that the mean methimazole dose required to maintain a euthyroid state during the first cycle of therapy, the initial remission rates and relapse rates following first methimazole cycle discontinuation, remission rates for at least 2 years following withdrawal of the last methimazole cycle, percentages of girls who underwent non-pharmacological therapies and definitive remission rates were similar between the two groups (29, 61) (Table 2).

## Trisomy 21 or Down Syndrome (DS)

DS is the most prevalent chromosomal abnormality affecting 1 in every 787 live births (63). Trisomy of chromosome 21 (non-disjunction) accounts for 95% of cases, while mosaicism or a Robertsonian translocation occurs in the remaining 5% of children (64). DS is associated with intellectual disability, growth retardation, congenital heart defects, gastrointestinal anomalies, increased risk of hematologic malignancies, hypotonia, hearing loss, ophthalmic and immunologic disorders, dysmorphic features and early-onset Alzheimer's disease (65, 66).

Furthermore, patients with DS exhibit increased susceptibility toward thyroid and non-thyroid autoimmune disorders, in particular alopecia and vitiligo, but also T1D, JIA and CD, the latter presenting more commonly among children older than 6 years of age (66–68) (Table 1). Plausible biological mechanisms explaining the predisposition to autoimmunity in DS cases may include: (1) thymic atrophy and diminished expansion of T and B lymphocytes in the first years of life. The T- lymphocyte subpopulation counts gradually normalize, whereas the B- lymphocytopenia persists. After the age of 6 years, DS children display a considerable hypergammaglobulinaemia of the IgA and IgG type, with increased levels of IgG1 and IgG3 and reduced levels of IgG2, IgG4, and IgM. Furthermore, a decrease in CD4+ along with an increase in CD8+ lymphocytes and the percentage of natural killer (NK) cells have been observed. Overall, these immunologic abnormalities may contribute to the increased risk of infections and autoimmune diseases in DS patients (64, 66, 69, 70) (2) altered thymic expression of the autoimmune regulator (*AIRE*) gene. This gene is located on chromosome 21q22.3, mainly expressed in thymic epithelial cells and encodes for a transcription factor that regulates the promiscuous expression of genes encoding tissue-specific antigens, thus playing a key role in the induction and maintenance of central tolerance by eliminating autoreactive T cells. Mutations in *AIRE* cause a rare autosomal-recessive disorder, autoimmune polyendocrine syndrome type 1 (APS-1), also called autoimmune polyendocrinopathy-candidiasis-ectodermal dystrophy (APECED) syndrome, with the cardinal features being autoimmune hypoparathyroidism, Addison's disease and chronic mucocutaneous candidiasis. Reduced intrathymic expression of AIRE accompanied by reduced expression of peripheral tissue-restricted antigens has been demonstrated in DS patients. Given that three copies of the gene were expressed in the thymus of those patients, the authors suggested that AIRE expression may be regulated by epigenetic or other post-transcriptional mechanisms in order to overcompensate for the excess of gene dosage (30). In contrast to the above findings, Skogberg et al. found increased expression of AIRE at both mRNA and protein level in the thymus of DS patients, implying that the increased *AIRE* gene dose in DS may alter thymic selection processes, thus contributing to autoimmune disease predisposition (71) (3) the genetic contribution of class II MHC genes. A strong association between the major histocompatibility complex (MHC) class II DQA 0301 allele and hypothyroid autoimmune thyroiditis was identified in DS patients, pointing toward a role of one or more genes on chromosome 21 that may regulate immune function (72) (4) altered activity of enzymes that modulate inflammatory and immune processes by regulating extracellular ATP and adenosine levels and the hydrolysis of acetylcholine (molecules involved in immune responses) resulting in increased pro-inflammatory cytokine levels such as IFN-γ, TNF-α, IL-1β, and IL-6, and decreased levels of anti-inflammatory cytokine IL-10 (73) (5) hyperresponsiveness to interferon (IFN) secondary to increased gene dosage of the four interferon receptors encoded on chromosome 21 (74, 75) (Table 1). In this regard, it should be pointed out that treatment with IFNa is associated with thyroid dysfunction via immune mediated and direct thyroid-toxic effects, which may be manifested as either autoimmune (HT or GD) or non-autoimmune destructive thyroiditis (76). Interestingly, *in vitro* studies have demonstrated that both INFα and -β inhibit the TSH-stimulated gene expression of thyroid peroxidase (TPO), sodium/iodide symporter (NIS), and thyroglobulin (TG) as well as T4 release (77). Taken together, it can be postulated that consistent activation of INF signaling may be implicated in the immune dysregulation and thyroid dysfunction associated with DS and may contribute to the increased susceptibility of DS patients to thyroid autoimmunity (74).

Thyroid dysfunction is the most frequently encountered endocrinopathy in DS, affecting 7 to 66% of patients. The spectrum of thyroid abnormalities includes congenital hypothyroidism (CH), isolated hyperthyrotropinemia or SH, primary hypothyroidism and thyroid autoimmunity, such as HT or GD (Table 1).

The incidence of CH is estimated between 1:113 and 1:141 DS live births (64, 66), being 28 times more common among DS patients compared to the general population, permanent in 70% and transient in 30% of cases (78, 79) (Table 1). Most cases are associated with thyroid hypoplasia, whereas thyroid agenesis, ectopy or goiter are infrequent. In line with the above, Luton et al. examined 13 fetuses with DS and reported that, the thyroid gland was eutopic in all fetuses, without gross anomalies, however, histologically, the thyroid follicles were abnormally small and heterogeneous in size. Moreover, they found TSH levels consistently above the 80th percentile, whereas the FT4 level was below the 50th percentile in most cases (80). Overall the pathogenetic mechanisms underlying CH in DS cases may include: (1) exaggerated response to thyroid-releasing hormone (TRH) stimulation until the third year of life and delayed maturation of the hypothalamic—pituitary—thyroid (HPT) axis (2) peripheral resistance to thyroid hormones resulting in inappropriate TSH secretion (3) inappropriate TSH release, due to a central defect or inadequate dopaminergic control (4) TSH insensitivity and reduced TSH bioactivity, which consequently may result in elevated TSH with low-normal T4 levels (64, 66). While the American Academy of Pediatrics (AAP) recommends screening for CH at birth and repeat TSH measurements at 6 and 12 months and annually thereafter, Pierce et al. have recently demonstrated that among those DS patients who were diagnosed with hypothyroidism beyond the newborn screening period, 11 (7.5% of all acquired hypothyroidism) were diagnosed before the age 6 months (81). Based on these findings, the authors recommended that TSH and FT4 be obtained at both 6–8 weeks and 4 months (81) (Table 2). Notably, it has been shown that thyroxine treatment within the first 2 years of life is associated with modest improvements in motor development and growth, however, without improvement of mental or motor development later in life. Nevertheless, it appears to have beneficial effects on growth outcome especially in children with neonatal plasma TSH concentrations higher than 5 mIU/L (78, 82, 83).

SH, defined as an elevated serum TSH level associated with normal total or free T4 and triiodothyronine (T3) values, is the most frequent thyroid abnormality among DS patients, with a prevalence ranging between 7 and 40% (64). The risk of developing thyroid dysfunction increases yearly by 10%, so that 25% of patients are expected to have thyroid disease by age 7.5 years and up to 50% by adulthood (81). On the other hand, SH before the age of 5 years may be a transient and self-limiting condition in >70% of DS cases. In this regard, the absence of goiter and thyroid autoantibodies have been associated with a greater chance of spontaneous remission (84). In the study of Pierce et al., euthyroidism was restored in 13% of DS patients with a history of hypothyroidism during follow-up, even in the presence of positive thyroid autoantibodies (81). It has been postulated that mild TSH elevation in DS may be an inherent defect of the syndrome reflecting a resetting of the HPT axis. Indeed, Meyerovitch et al. revealed that the 2.5th to 97.5th percentile TSH values in a cohort of DS patients ranged between 1.3 and 13.1 mIU/l, with the 95th percentile value being 8.9 mIU/l, i.e., significantly higher compared to controls. A similar upward shift was also shown in the distribution of FT4 levels (85). For these reasons, it has been supported that SH may be overdiagnosed in DS patients, leading to unnecessary life-long treatment. In addition, SH has not been shown to be a precursor of permanent hypothyroidism (i.e., low T4, increased TSH levels) and the 10 year incidence for onset of definite hypothyroidism has been reported to be low, 13.6% (86).

From another point of view, L-thyroxine treatment for SH in DS patients, whose development and growth are already inherently substantially compromised, is considered a safe and inexpensive treatment with possible benefits on growth and intellectual outcomes, if special attention can be laid to avoid pharmacological hyperthyroidism (64, 66, 82). Moreover, it has been shown that thyroxine treatment during the first 2 years of life was associated with higher FT4 concentrations at age 10.7 years, indicating that early thyroxine treatment may result in an alteration in the set point of the HPT axis that may persist later in life. In addition, the frequency of positive anti-TPO antibodies increased with age from 1% at the age of 12 months, to 6% at age 24 months and 25% at age 10.7 years in the DS placebo group, whereas there was a trend toward a lower percentage of children developing anti-TPO positivity among DS patients who received thyroxine treatment. The above results may indicate a protective effect of early levothyroxine treatment, possibly by reducing TSH levels and thus thyroid autoantigen presentation (87).

Taken together, in cases of mild TSH elevation, repeat testing of FT4 and TSH levels in a 2–3 month time, along with measurement of thyroid autoantibodies titers, should be considered in order to avoid treatment initiation in SH-cases that may be proved transient. With regards to thyroid antibodies screening, the Ireland and UK guidelines recommend testing (including T4 and TSH levels) at least once every 2 years, from age 1 year and throughout life (88). The presence of positive antithyroid antibodies alone does not warrant treatment initiation but TSH and FT4 levels should be monitored regularly in order to early detect conversion from euthyroidism to hypothyroidism. Treatment with levothyroxine is advisable in DS patients with SH and a) TSH levels >10 μU/ml b) TSH levels <10 μU/ml in the presence of goiter and/or positive thyroid antibodies. Moreover, special considerations should be given in infants and children below the age of 5 years. Treatment should be initiated in all cases that progress to overt hypothyroidism (low FT4 and elevated TSH levels) or become symptomatic. A trial off therapy and re-evaluation of thyroid function tests should be offered in those patients who do not require increase in their levothyroxine dose following treatment initiation or displayed no rise of TSH >10 μU/ml while on treatment. Regular auxological and clinical assessment and life-long monitoring of the thyroid function are recommended for all patients (64, 66, 78, 81, 84, 89) (Table 2).

Autoimmune hypothyroidism is the most frequent autoimmune disorder in DS. Antithyroid antibodies are found in 13–46% of patients (Table 1). Interestingly, almost 50% of them, develop thyroid antibodies positivity at an age younger than 8 years (64, 81). In contrast to the general population, HT in DS is characterized by the following features: (1) younger age at diagnosis (mean: 6.5 years), possibly reflecting the increased awareness of physicians on DS-associated thyroid dysfunction which may result in an earlier detection of HT cases among DS patients (2) lower frequency of positive family history of thyroidopathy and higher frequency of extra-thyroidal autoimmune diseases, suggesting that DS is *per se* associated with an increased risk of developing autoimmune disorders (3) no gender predilection (4) lower prevalence of euthyroidism and increased prevalence of SH at presentation, despite lower anti-TG and anti-TPO levels, suggesting probably a congenital alteration in thyroid gland regulation (5) progressive deterioration of thyroid function over time (median interval of 5.1 years) as the prevalence rates of overt hypothyroidism and hyperthyroidism were shown to increase, whereas the frequency of SH remained unchanged (6) more frequent evolution toward GD (64, 66, 68, 90, 91).

GD occurs more frequently in DS children compared to the general population with an estimated prevalence of 6.5 vs. 1.07%, respectively (68, 92) (Table 1). Compared to patients without DS, GD in affected subjects usually presents at a younger age, between late childhood and early adulthood, is more frequently associated with other autoimmune diseases, namely CD, presents a higher rate of positive family history of HT but no gender predominance (92, 93). Furthermore, as already mentioned above, the risk of conversion from HT to GD is higher (64, 90). In the study of Aversa et al., long-term remission could be achieved in all GD patients following the onset of antithyroid medication. Low doses of methimazole treatment were required to maintain euthyroidism, no relapses occurred after treatment withdrawal and no alternative treatments were needed, pointing toward a less severe clinical course of GD in DS (90). Similar findings have been previously reported in a multicenter Italian study by De Luca et al., comprising 28 DS children with GD and 109 children and adolescents with GD but without DS (control group) (93). Lower relapse rates after the first cycle of methimazole treatment withdrawal and higher persistent remission rates after definitive methimazole withdrawal were observed in DS patients compared to controls. In addition, no DS patient required surgery or radioiodine ablation, compared to 11% of controls who underwent non-pharmacological therapies. Of note, a longer time to achieve remission, but also a trend toward higher remission rate could be observed in GD patients with DS compared to those without in a more recent study (94). Furthermore, none of the DS patients received definitive therapy, compared to 36% of those without DS. On the contrary, Goday-Arno et al. reported that no patient could achieve remission following carbimazole discontinuation in their study population. Radioactive iodine was administered after a mean period of medical treatment of 40 months without clinical remission. Hypothyroidism developed in all treated cases necessitating replacement therapy with levothyroxine (92). At this point it should be noted, that despite the reluctance to offer radioiodine treatment in pediatric patients with GD as second-line therapy, surgery may not be an optimal choice for DS patients with the disease, as their short necks, craniofacial anomalies and associated airway obstruction may predispose to increased anesthetic and surgical risks (64, 92) (Table 2).

Of interest, in the study of Aversa et al., one third of GD patients shifted from hyper- to hypothyroidism following methimazole discontinuation. It should be stressed, however, that all DS children enrolled in this study had a previous diagnosis of HT. It can be postulated that the HT-related damage of the thyroid might have overcome the stimulating effects of the TSHR autoantibodies, resulting, thus, in the aforementioned conversion from hyper- to hypothyroidism in a proportion of patients (90).

## Klinefelter Syndrome

Klinefelter syndrome (KS) is the most common chromosomal aberration in males, affecting 1 in every 660 men (95). It results from meiotic or mitotic non-disjunction, leading to the presence of one or more extra X chromosomes. Most patients (90%) have the classic 47,XXY karyotype, whereas higher-grade aneuploidies (48,XXXY; 49,XXXXY), structurally abnormal X chromosome (e.g., 47,iXq,Y) or mosaicisms (47,XXY/46,XY) account for the remaining 10% of cases (96). It is characterized by broad phenotypic variability with regards to physical traits, cognitive abilities and comorbidities and remains remarkably underdiagnosed with only around 25–39% of cases receiving a diagnosis postnatally and <10% before puberty (97–99).

Hypergonadotropic hypogonadism, gynecomastia, small firm testes, infertility, sparse facial and pubic hair, tall stature along with impaired psychosocial functioning of variable degree are common features of the condition (99). Moreover, KS is associated with multiple comorbidities, including cardiovascular, cerebrovascular and thromboembolic diseases, osteoporosis, diabetes mellitus, metabolic syndrome, as well as an increased risk of developing breast cancer and extragonadal germ cell tumors (96, 100). In addition, recent studies have demonstrated that KS patients are at increased risk of certain autoimmune disorders, in particular Addison's disease, T1D, multiple sclerosis, acquired hypothyroidism, rheumatoid arthritis, Sjogren's syndrome and systemic lupus erythematosus (SLE), most of which being female predominant (101). Concurrence of KS with other inflammatory rheumatic diseases such as JIA, psoriatic arthritis, polymyositis/dermatomyositis, systemic sclerosis, mixed connective tissue disease, antiphospholipid syndrome, ankylosing spondylitis and primary biliary cirrhosis has been also reported (102) (Table 1). Endocrine organ-specific autoantibodies can be detected in 13% of KS subjects and the frequency progressively increases in those with higher-grade aneuploidies and is higher in children than in adults (103). It is worth noting, that KS patients exhibited autoantibodies primarily (i.e., 8.2%) against diabetes-specific autoantigens (104).

Positive thyroid antibodies are found in 5.4–10% of children and 3.3–7% of adults with KS in different studies (103–106). Most cases are euthyroid, whereas GD occurs only rarely in patients with the syndrome (107, 108) (Table 1).

The influence of sex hormones as modulators of the immune response and X-linked gene dosage have been implicated in the pathogenesis of autoimmunity in KS (Table 1). The immunostimulatory effects of estrogens and the immunosuppressive effects of androgens are well-established. In males with rheumatoid arthritis both low testosterone and increased estradiol have been observed, with the latter being correlated with the degree of inflammation (109, 110). On the other hand, patients with KS often display elevated estradiol levels, which are comparable to those seen in normal menstruating women and androgen levels like those of a preadolescent male (111). Low serum testosterone levels were also found in males with SLE and a recent record linkage study demonstrated an association between testicular hypofunction and SLE (112, 113). Of interest, previous studies on sex hormone metabolism in KS patients with SLE revealed that this was similar to the one seen in women affected by SLE (114). Taken together, the above findings highlight the potential role of sex hormones as contributors to the development of autoimmunity in patients with KS (Table 1).

Notably, the risk of SLE among KS patients is 14-fold increased compared to 46,XY males and similar to the risk seen in 46,XX females (115), pointing toward the role of the number of X chromosomes and in particular an X-linked gene dose effect (Table 1). Indeed, genes escaping X inactivation may have higher levels of expression in subjects with two X chromosomes (116). The *CD40 ligand* gene, located in the Xq26.3 region, encodes a protein expressed on the surface of activated T cells, which binds to CD40 on the B cell surface and mediates B cell proliferation and immunoglobulin isotype switching, thus playing an important role in adaptive immunity (117). *In vitro* studies reported by Sarmiento et al. showed higher percentage of CD40L-expressing CD3+ T cells and CD40L protein and mRNA expression after activation in females and KS patients compared to males and TS women, indicating that the existence of two copies of the X chromosome may enhance both cell-mediated and humoral immune responses (118). Another gene, *TLR7*, mapped to Xp22.2, encodes a member of the Toll-like receptor (TLR) family, which detects single stranded-RNA and activates innate immune responses, such as the production of inflammatory cytokines and type I interferons (119). Sarmiento et al. demonstrated also higher Toll-like receptor 7 (TLR7) mRNA levels post-stimulation in healthy females and KS patients than in TS women and males. However, TLR7-mediated IFN-alpha production did not differ between KS patients and healthy males, suggesting that hormonal factors or epigenetic alterations may modulate the innate immune response (118).

Apart from autoimmunity, other mechanisms involved in the development of thyroid dysfunction in KS patients include some degree of central hypothyroidism and peripheral hypothyroidism. A low TSH response to TRH stimulation has been previously observed in KS patients and attributed most likely to the chronic compensatory increase in the production of gonadotropins, leading to reduced availability of the alpha subunit for the formation of TSH (120, 121). Moreover, KS patients displayed lower FT4 and FT4/free T3 (FT3) ratio than control men with no increase in their serum TSH, whereas their FT4 values were in or just below the lower limits of the normal range. The above findings may suggest an impaired hypothalamic-pituitary control of the thyroid function resulting in secondary thyroid insufficiency (120). Similar TSH but lower FT4 levels were also documented in KS patients when compared to non-KS hypogonadal men with similar testosterone levels (105). Further to the above, pubertal KS patients showed similar FT4 and TSH, but lower FT3 levels when compared to age-matched pubertal healthy boys, indicating a mixed form of hypothyroidism in KS boys during the pubertal development: both secondary and peripheral, the latter due to reduced deiodinase activity (106) (Table 1). Interestingly, a Danish study aiming to explore the association between KS comorbidities and differential gene expression profiles, revealed that genes involved in ‘abnormal thyroid hormone metabolism’ were upregulated in KS patients (122).

Taken into account the above data and given the lack of formal recommendations on screening and monitoring of thyroid function in KS, we suggest measurement of TSH and FT4 levels at diagnosis and annually thereafter. Screening for thyroid autoantibodies should be considered in case of TSH elevation and/or presence of goiter or periodically in the absence of suggestive clinical or biochemical signs (Table 2).

## 22q11.2 Deletion Syndrome

Chromosome 22q11.2 deletion syndrome (22q11.2DS) is the most common microdeletion syndrome with an estimated incidence of 1:4.000 live births worldwide. 22q11.2DS encompasses a heterogeneous group of phenotypically similar disorders, including DiGeorge syndrome (DGS), velocardiofacial syndrome (VCFS), conotruncal anomaly face syndrome (CTAF), some cases of autosomal dominant Opitz G/BBB syndrome and Cayler cardiofacial syndrome. It is caused by a (1.5–3.0 Mb) hemizygous deletion at chromosome 22q11.2, which is *de novo* in more than 90% of cases and inherited from a heterozygous parent in ~10%. The cardinal features of the condition are congenital cardiac defects, mainly conotruncal malformations (ventricular septal defect, tetralogy of Fallot, interrupted aortic arch and truncus arteriosus), palate anomalies, thymic hypoplasia and immunodeficiency, neonatal hypocalcemia, developmental delay, learning disabilities and dysmorphic facial features (123, 124).

Endocrinopathies are identified in 60% of patients (125). In particular, hypoparathyroidism and hypocalcemia are observed in 17–60%, thyroid gland anomalies in 50% and hormonal dysfunction in up to 25.6%, short stature in 41%, growth hormone deficiency in 4%, whereas obesity is present in up to 43.5% of adults with the syndrome (123, 126–128).

Furthermore, 22q11.2DS patients are prone to develop autoimmune disorders, the most common being ATD, JIA, and ITP (129). Coexistence of autoimmune haemolytic anemia, autoimmune neutropenia, psoriasis, vitiligo, CD, pernicious anemia/atrophic gastritis, inflammatory bowel disease, urticaria, Raynaud phenomenon, adult RA, rheumatic fever with chorea and T1D in 22q11.2DS patients has been also reported (129–133) (Table 1). Of interest, in the study of Lima et al., 9 of 28 22q11.2DS patients (32%) tested positive for adrenal autoantibodies, however ACTH and cortisol levels were normal in all cases (133). Overall, autoimmune phenomena may occur in 10–30% of affected individuals (129–131, 133).

ATD is common among individuals with 22q11.2DS. Thyroid autoantibodies were found in up to 5% of affected children and 30% of patients older than 17 years (127, 129, 133, 134) (Table 1). In the study of Shugar et al., overt thyroid disease was noted in 9.5% of 169 children with the syndrome. Hypothyroidism occurred in 7.7% and hyperthyroidism in 1.8% (Table 1). Interestingly, among patients with thyroidopathy the female to male ratio was 2.2, which is lower than the one reported in the general pediatric population. Furthermore, of those with prodromal or subclinical thyroid disease, 42% progressed to overt thyroid disease within a mean follow-up time of 27.6 months, necessitating medical treatment (135). In a previous study conducted in adults with 22q11.2DS the frequency of hypothyroidism reached 20.5% (136).

The association between GD and 22q11.2DS is noteworthy. As mentioned above, the prevalence of hyperthyroidism in the pediatric 22q11.2DS population (1.8%) is far higher than in the general pediatric population (1 in 10.000 children in the United States) (135, 137) (Table 1). Moreover, atypical presentation with seizures and very young age at onset (as early as 27 months of age) have been described in pediatric cases of 22q11.2DS, features that are quite unusual especially when taking into account that GD rarely occurs in children younger than 5 years of age (127, 137–139). Hyperthyroidism may occur at any age and affects around 5% of adults with the disorder (136).

A complex interplay between immunologic, genetic, and environmental factors may underlie the association between GD and 22q11.2DS. Possible mechanisms may include the following: (1) Absent or hypoplastic thymus may result in impaired maturation and dysregulation of T cells and thus defective central tolerance allowing self-reactive T cells to escape intrathymic negative selection and trigger autoimmune responses (124). In this regard, abnormal thymic development may lead to decreased AIRE expression and consequently impaired AIRE-mediated intrathymic expression of tissue-restricted antigens (TRAs) (140). (2) Certain HLA haplotypes, such as HLA-DR14 may increase susceptibility to GD in patients with 22q11.2DS (139, 141). (3) Thymic maldevelopment may result in several immunologic abnormalities. T-cell lymphopenia and decreased absolute counts of CD4+CD25+ natural Tregs have been observed in 22q11.2DS patients especially during the first 4 years of life and might explain the early age of GD onset in some cases (142–144). (4) Furthermore, lymphopenia and restricted T cell repertoires, abnormal T cell activation and associated B cell dysregulation and humoral dysfunction, as well as Th1/Th2 imbalance of the CD4+ T cells may also be implicated in the induction of autoimmunity (130, 141, 145, 146) (Table 1).

Further to ATD, thyroid gland anomalies can be also more commonly been observed in 22q11.2DS patients (Table 1). Among 28 22q11.2DS children who underwent computed tomography (CT) scans 50% exhibited thyroid gland abnormalities, including an absent isthmus (21%), retrocarotid (14%), and retroesophageal extension (14%) and absence of the left thyroid lobe (one case). Elevated TSH levels were detected in 2/14 patients. Low common carotid artery bifurcations were also noted (147). In a subsequent study, Stagi et al. implied that thyroid hypoplasia may be a feature of 22q11.2DS, given that 46.6% of 30 patients studied had decreased total thyroid volume, with the left thyroid lobe being significantly smaller in all cases. Overt and subclinical hypothyroidism were found in 3.3 and 23.3% of all study population, respectively, and thyroid hypoplasia was noted in the majority of these cases (62.5%). Of note, 71% of patients who presented with thyroid hypoplasia had coexistent congenital heart malformation, compared to 31% of those with a normal thyroid volume (148). Deeper insights into mechanisms involved in the pathogenesis of 22q11.2DS unraveled the major role of T-box transcription factor *(TBX1)* gene. *TBX1*, mapped on the long arm of chromosome 22 at position 11.21, encodes a transcription factor protein essential for the regulation of developmental processes in a number of tissues, derived from the pharyngeal apparatus, such as the development, positioning and size of thyroid gland. Interestingly, Tbx1–/– mice exhibited most of the cardiac and pharyngeal arch anomalies seen in the 22q11DS, including cardiac outflow tract abnormalities, thymic and parathyroid gland hypoplasia and craniofacial anomalies (147–149).

The above findings underline the importance of a meticulous clinical and endocrine evaluation of all 22q11.2DS patients at diagnosis and during follow-up in order to address and appropriately manage associated comorbidities. With respect to thyroid function, baseline screening should include measurement of TSH, FT4, total T3, and TPO autoantibodies. If the results are normal, thyroid function tests (TFTs) should be checked on an annual basis lifelong. When overt disease is biochemically confirmed, appropriate medical treatment should be started. In case of subclinical thyroid disease, thyroid function tests (TFTs) should be repeated in a 4–6 months' time interval. If subclinical disease persists (and in case of overt thyroid disease), a thyroid ultrasound should be performed in order to exclude structural anomalies of the gland (135). Medical treatment for subclinical disease should be offered if TSH levels remain abnormal despite normal FT4 levels in the presence of suggestive signs/symptoms and/or positive TPO antibodies and/or goiter or thyroid hypoplasia. Alternatively, TFTs can be rechecked after another 4–6 months' time interval (Table 2).

## Williams Syndrome

Williams–Beuren or Williams syndrome (WS) is a multisystemic neurodevelopmental disorder caused by a 1.5–1.8 Mb heterozygous microdeletion at chromosome 7q11.23, encompassing 26 to 28 genes. It is a rare disorder, affecting roughly 1 in 10.000 people. Common clinical manifestations include intellectual disability, a constellation of dysmorphic facial features known as elfin facies, connective tissue abnormalities and cardiovascular disease, namely supravalvular aortic and pulmonary artery stenosis. In particular, haploinsufficiency of elastin *(ELN)* gene, located in the deleted region is responsible for the cardiovascular abnormalities of the disorder, whereas hemizygosity of LIM Domain Kinase 1 *(LIMK1)*, mapped to the same area, may contribute to the cognitive impairment associated with the syndrome (150–152).

Endocrine disorders are frequently encountered in patients with WS, including short stature, hypercalcemia, central precocious puberty, hypothyroidism, impaired glucose tolerance and diabetes, dyslipidemia, decreased pubertal growth spurt, GH deficiency, decreased bone mineral density or osteoporosis (150, 153–155).

SH occurs in 15–37.9% of patients, is commonly associated with thyroid hypoplasia and its prevalence decreases with age (156–159) (Table 1). In this regard, the frequency of SH in WS children was considerably higher in those younger than 3 years (73.9%) compared to older patients, whereas the vast majority of children aged more than 9 years were euthyroid (158). Selicorni et al. noted the highest incidence of SH in patients under 1 year of age (157). Similarly, Chen et al. demonstrated that the frequency of SH in WS increased during the first years of life, reaching 44.4% among children aged 3–6 years, and declined gradually thereafter (159). The above findings may reflect immaturity of the HPT axis, further supported by an exaggerated, prolonged TSH response to TRH as well as a mildly low biological activity of circulating TSH, which were observed in a 2 month old female infant with WS, thyroid hemiagenesis and elevated TSH levels (160). TSH insensitivity of the thyroid gland has been also proposed to explain HPT axis dysfunction in affected individuals (159). Notably, patients with WS rarely present CH (Table 1). However, this could be the initial manifestation that could raise suspicion for the syndrome, in the presence of suggestive clinical features (161, 162).

With regards to the natural history of SH in WS, 13% of patients with normal thyroid function at baseline showed abnormal TSH levels at follow-up. On the contrary, 70% of those who had initially elevated TSH levels, were euthyroid on follow-up evaluation (159). These data point toward a transient, self-limiting condition in most cases, like the one observed in DS patients and the general pediatric population, as well. Overt hypothyroidism is less common (10%) (156) and thyroid autoantibodies are negative in most cases (156–159) (Table 1).

On the other hand, thyroid hypoplasia is a frequent feature of WS, presenting in up to 75% of patients (156, 157), with the left lobe being more commonly affected (156). Other structural abnormalities of the thyroid gland may include agenesis, hemiagenesis and ectopy (151, 156, 160, 163) (Table 1). From a biochemical point of view, most patients with hypothyroidism had thyroid hypoplasia, indicating that thyroid dysfunction in WS may result from reduced thyroid volume (156, 157). Taken into consideration that the prevalence of SH decreases with age, it can be assumed that the hypoplastic thyroid gland cannot compensate for the increased requirements for thyroid hormones during the very first years of life, resulting in TSH elevation. However, when the need for thyroid hormones decreases with age, the hormonal production by the hypoplastic gland may be sufficient leading to normalization of TSH levels in most cases (157).

Furthermore, thyroid hypoplasia was more frequently seen in older children, pointing toward poor growth of the thyroid gland, possibly associated with genetic defects within the 7q11.23 region (156, 158, 159). Indeed, *BAZ1B* (Bromodomain Adjacent to Zinc Finger Domain 1B), located in the deleted WS-region, has been recently implicated in the thyroid gland developmental defects seen in a percentage of patients with the syndrome. It encodes a tyrosine-protein kinase, which functions as a transcription regulator and plays a key role in chromatin remodeling. The most recent study by Allegri et al. demonstrated that BAZ1B silencing results in reduced cell viability and survival of human thyroid cells, largely due to an increase in apoptotic phenomena and these effects may be mediated through phosphatase and tensin homolog PTEN overexpression (164).

Apart from primary hypothyroidism, other thyroid function abnormalities are rare. In the study of Amenta et al., comprising 50 patients with WS, hyperthyroidism occurred in one case (152). Interestingly, central hypothyroidism and secondary adrenal insufficiency were most recently reported in a 10 month-old boy with WS (165) (Table 1).

With regards to concurrent autoimmune disorders, as already mentioned, thyroid autoantibodies are rarely present in patients with WS (152, 166). Of note, Giannotti et al. reported an increased prevalence of CD in WS subjects (9.5% vs. 0.54% in the general pediatric population in Italy) (31) (Table 1), whereas Stagi et al. found no evidence of increased autoimmunity in their patients with WS, including CD (32).

The American Academy of Pediatrics currently recommends thyroid function evaluation in WS patients at a minimum: at diagnosis, annually for the first 3 years and at 2 years' intervals thereafter (167). However, in light of the above findings concerning the high frequency of thyroid structural abnormalities associated with WS as well as the higher prevalence of thyroid dysfunction in early childhood, it would be prudent to suggest assessment of both thyroid function and morphology as soon as the diagnosis of WS is made and even if the newborn screening result for CH is negative. Moreover, in patients younger than 6 years, or in those with thyroid hypoplasia, close monitoring of the thyroid function in shorter intervals, i.e., at 3–6 months from baseline, and then yearly, should be considered. Given the higher prevalence of thyroid hypoplasia in older patients, thyroid ultrasound should be performed regularly until adulthood. In case of overt hypothyroidism treatment with levothyroxine should be promptly initiated. SH may be a self-remitting condition, so that ongoing monitoring without treatment initiation may be considered. However, levothyroxine replacement therapy is advisable, especially in children younger than 3 years, if TSH levels are persistently elevated, in the presence of thyroid hypoplasia and/or suggestive signs and symptoms (Table 2). In addition, considered the increased cardiovascular risk associated with WS, treatment could be likely of benefit for some of these patients in order to prevent further cardiovascular impairment (157, 159).

## Prader-Willi Syndrome

Prader-Willi syndrome (PWS) is a genomic imprinting disorder due to the lack of expression of paternally inherited genes within the chromosome region 15q11-q13. *De novo* paternally derived deletions of the chromosome 15q11-q13 region account for around 75% of cases, maternal uniparental disomy (UPD) for 24% and defects in the genomic imprinting center, chromosomal translocations or rearrangements within 15q11.2-q13 region for 1–3%, respectively. It is considered to be the most frequent form of syndromic obesity, affecting 1:10.000 to 1:30.000 people (168–170). The major clinical features of the disorder include neonatal hypotonia with feeding problems and failure to thrive during infancy, followed by excessive eating and rapid weight gain in early childhood, accompanied by global developmental delay and behavioral problems. Short stature, distinctive facial appearance, small hands and feet, cryptorchidism, and scoliosis are also common findings (169–171). Hypothalamic dysfunction may underlie many components of the syndrome, including hyperphagia, sleep abnormalities, temperature dysregulation, and multiple pituitary hormone insufficiencies, including growth hormone (GH), adrenocorticotropic hormone (ACTH), TSH deficiencies, hypogonadotropic hypogonadism, but also, less commonly, precocious puberty (170, 172, 173). Pituitary gland abnormalities have been reported in more than 60% of cases regardless of the presence or number of pituitary deficiencies. Those may include empty sella, pituitary hypoplasia, small, globular, or displaced posterior pituitary gland or complete absence of the posterior pituitary gland bright spot (172, 173).

The prevalence of hypothyroidism in PWS, mainly of central origin, varies widely from 2.1 to 32%, with only few studies addressing this issue (172–180). In a recent study comprising 339 individuals with PWS (71.7% aged below 18 years), the frequency of thyroid dysfunction was 13.6%: 6.8% had central hypothyroidism (of whom 43.5% were younger than 1 year), 3.8% subclinical hypothyroidism, 1.8% hypothyroidism and 1.2% CH (179) (Table 1).

Vaiani et al. examined the thyroid function in infant PWS patients, revealing a frequency of 72.2% of central hypothyroidism during the first 2 years of life. Moreover, patients with thyroid dysfunction displayed lower body length which correlated positively to FT4 levels, indicating that early onset hypothyroidism may impair normal growth (175). In contrast, studies conducted in older PWS patients reported a far lower prevalence of hypothyroidism (172–174, 176, 177), indicating that the thyroid dysfunction observed at very young PWS children might be associated with delayed CNS development and normalize with further CNS maturation, as these patients get older (175). Despite low T4 and/or FT4 levels in most patients in the study of Vaiani et al., T3 levels were low in just one case. This finding indirectly suggests, that low FT4 levels may be compensated for by an increase in peripheral conversion of T4 to T3 (175). Of note, leptin, produced by adipose tissue in proportion to its mass, increases deiodinase type 2 activity. Interestingly, young, still underweight children were shown to have relatively increased body fat and elevated BMI-adjusted leptin levels (181). In the study of Sharkia et al., PWS children had FT4 levels in the lower half and FT3 above the median of the normal range, findings that could be interpreted within the same context (176). GH replacement therapy may also contribute to the decrease in FT4 concentrations, reflecting either a central inhibition of TSH release due to increased somatostatinergic tone or increased peripheral conversion from FT4 to T3, which could further lead to T3 negative pituitary feedback (178, 180).

Notably, concurrent CH with fetal goiter or due to ectopic, sublingual thyroid gland has been described in association with PWS (182–184) (Table 1). Taken into account that signs and symptoms of hypothyroidism and PWS may overlap, the diagnosis of PWS should be considered in cases with CH and severe infantile hypotonia that do not improve, despite appropriate levothyroxine replacement therapy.

Given that endocrine dysfunction in PWS is mainly of hypothalamic origin, thyroid autoantibodies were measured in none but one study, reporting slightly positive TPO antibodies in one out of 21 PWS patients (176). Of interest, autoantibodies against pituitary (APA) were recently detected in 30.9% of 55 PWS patients, a frequency far higher than in healthy controls (Table 1). In addition, APA were more common in those with uniparental maternal disomy for chromosome 15 than in those with interstitial deletion of the proximal long arm of paternal chromosome 15. Based on these findings, it can be postulated that autoimmune processes may be involved, at least in part, in the pituitary impairment associated with PWS. However, given that the rate of positive APA did not differ among those with and without pituitary deficiencies, their clinical significance remains to be determined (185).

Based on published reviews and expert recommendations, TSH and FT4 levels should be measured within the first 3 months of life, regardless of the newborn screening result, taken into account that TSH-based screening strategies cannot detect central hypothyroidism. Subsequently, TSH and FT4 should be determined annually, with consideration of more frequent testing if the patient receives treatment with rGH. Appropriate thyroid hormone replacement therapy should be initiated, if indicated. Taken into account the higher frequency of hypothyroidism during the first 2 years of life, as well as, the detrimental effects of untreated hypothyroidism for somatic growth and development, that could be further compromised in PWS patients, particular attention should be paid in infancy and early childhood, to ensure adequate thyroid hormone levels (168, 171, 178) (Table 2).

## Rasopathies: Noonan Syndrome and Neurofibromatosis Type 1

The RASopathies are a group of neurodevelopmental syndromes caused by germline mutations in genes encoding protein components or regulators of the Ras/mitogen-activated protein kinase (MAPK) pathway, a ubiquitous signaling transduction pathway activated by a large number of extracellular stimuli (growth factors, hormones, cell/cell interaction) to regulate essential cellular functions, such as proliferation, survival, differentiation, migration, or metabolism. Ras proteins are small guanosine nucleotide-bound GTPases activated following binding of a growth factor to receptor tyrosine kinases (RTKs), G-protein-coupled receptors, cytokine receptors, and extracellular matrix receptors. They alternate between a guanosine diphosphate–bound inactive state to a guanosine triphosphate (GTP)–bound active state. Ras-GTP can activate several downstream effector pathways namely, phosphatidylinositol 3-kinase (PI3K), RAL guanine nucleotide exchange factor and the RAF kinases, the first MAPK kinase of the pathway. RAS-MAPK signaling dysregulation has profound pathophysiological consequences. Somatic mutations of genes encoding RAS-MAPK components resulting in RAS/MAPK pathway hyperactivation have been found in several types of cancer, whereas germline mutations are causally linked to RASopathies. This term designates a group of clinically related disorders sharing overlapping clinical features, including craniofacial dysmorphism, short stature, cardiac malformations, cutaneous lesions, musculoskeletal, and ocular abnormalities, neurocognitive dysfunction of variable degree, and an increased risk of cancer. RASopathies affect ~1 in 1,000 live births and include the following conditions (and associated gene mutations): Neurofibromatosis type 1 (NF1) (*NF1* gene), Noonan syndrome (NS) (activating mutations in *PTPN11, SOS1, RAF1, KRAS, NRAS, SHOC2, CBL*), Noonan syndrome with multiple lentigines (NSML) (*PTPN11, RAF1*), capillary malformation–arteriovenous malformation syndrome (CM-AVM) (haploinsufficiency of *RASA1*), Costello syndrome (CS) (activating mutations in *HRAS*), cardio-facio-cutaneous syndrome (CFC) [activating mutations in *BRAF, MAP2K1* (*MEK1*) or *MAP2K2* (*MEK2*)], and Legius syndrome (inactivating mutations in *SPRED1*) (186, 187).

NS is a multisystem genetic disorder characterized by dysmorphic craniofacial features, including a broad forehead, hypertelorism with down-slanting palpebral fissures, ptosis, low-set posteriorly rotated ears with a thickened helix, short or webbed neck, congenital heart disease (most commonly pulmonary valve stenosis, hypertrophic cardiomyopathy and atrial septal defects), short stature, chest deformities, lymphatic dysplasias, ocular abnormalities, cryptorchidism, learning difficulties, short stature, renal anomalies, hearing loss and developmental delay of variable degree (188–190). Furthermore, affected patients have an 8-fold increased risk of developing childhood cancer, including juvenile myelomonocytic leukemia, acute myelogenous leukemia, B-cell acute lymphoblastic leukemia, whereas cases with solid tumors, such as rhabdomyosarcoma and neuroblastoma have been also reported (190–192). NS occurs in 1:1000 to 1:2500 live births (190) and is the second most frequent syndromic cause of congenital heart disease after Down syndrome (190, 191). The diagnosis is primarily based on clinical grounds, however causative gene mutations can be identified in around 70% of cases (193). The disorder follows mostly autosomal dominant inheritance with a near complete penetrance but a considerable variable expressivity (188, 194). Sixty percentage of affected individuals have *de novo* mutations (193). Around 50% of patients harbor mutations in the *PTPN11*, 10–15% in the *SOS1*, 3% in the *KRAS* and 3–15% in the *RAF1* genes, enhancing the function of the RAS/RAF-MAPK pathway (190). *PTPN11* (tyrosine-protein phosphatase non-receptor type 11) encodes for the non-receptor protein tyrosine phosphatase SHP-2 (Src homology phosphotyrosyl phosphatase 2). The majority of PTPN11 missense, gain-of-function mutations associated with NS primarily impair the activation/inactivation molecular switch of SHP2, resulting in constitutive or prolonged activation of the protein and increased activation of the Ras/MAPK pathway (186, 191).

Endocrine disorders associated with NS include short stature, growth hormone deficiency, neurosecretory dysfunction, and GH resistance, delayed puberty, diminished pubertal growth spurt, cryptorchidism and male gonadal dysfunction (191, 194). The frequency of thyroid autoantibodies in NS has been reported in a few studies, varying between 14.3 and 60%, whereas hypothyroidism occurs less commonly (4–21.4%) (195–199). In the study of Quaio et al., comprising 42 patients with RASopathies, the majority of whom had NS, 17% had subclinical hypothyroidism without thyroid antibodies, 7% were euthyroid having positive thyroid autoantibodies and another 7% presented overt autoimmune hypothyroidism (199) (Table 1).

On the other hand, in the study of Svensson et al., the frequency of thyroid antibody positivity did not differ between children with NS and controls. However, it tended to increase at puberty and with age in NS patients. In particular, 30% of those aged 12 years or older but none among those younger than 12 years had positive thyroid autoantibodies (195), suggesting that testing for thyroid autoantibodies should be performed periodically during adolescence in patients with NS.

Interestingly, accumulated evidence points toward a possible link between NS and autoimmune disorders. Scattered cases with NS and coexistent vasculitis, vitiligo, thyroiditis, anterior uveitis, SLE, CD have been anecdotally described in the literature (196, 200, 201). More recently, Quaio et al. revealed the presence of autoantibodies in 52% and autoimmune diseases in 14% of 42 patients with RASopathies, 37 of whom had NS. Those included autoimmune thyroiditis, SLE, polyendocrinopathy (association of autoimmune thyroiditis and CD), antiphospholipid syndrome, vitiligo, and autoimmune hepatitis and all occurred in patients with *PTPN11* mutations (199) (Table 1). At this point, it is worth mentioning, that one of the SLE susceptibility loci has been identified at 12q24, a locus encompassing *PTPN11*, germline mutations of which are found in 50% of NS cases. The linkage of a susceptibility gene for SLE to a region implicated in NS lends further support for a possible association between NS and SLE (199, 200, 202).

In this regard, the activity of SHP2 (the protein product of *PTPN11*) was shown to be increased in both lupus-prone mice and in human SLE patients. Of interest, inhibition of SHP2 activity diminished skin lesions, increased life span, reduced lupus-associated organ damage, blocked abnormal T cell proliferation, normalized extracellular signal regulated kinase ERK/MAPK signaling, and decreased production of IFN-γ and IL-17A/F in lupus-prone mice. In addition, SHP2 inhibition reduced the proliferation of cultured human lupus T cells and decreased the production of IFN-γ and IL-17A/F *in vitro*, suggesting integral involvement of SHP2 in human lupus-associated immunopathology (203). Moreover, SHP-2 inhibits activation of human NK cells upon recruitment to killer cell Ig-like receptors (KIR), but may also inhibit both cytolytic activity and IFN-γ secretion by NK cells independently of its role in KIR signaling (204, 205). SHP-2 also functions as a regulator of NF-κB/transcription factor nuclear factor κB activation, which in turn plays a critical role in various biological processes, including immune response, inflammation, cell survival and oncogenesis (206). Given that NK cells and NF-κB are important components of the immune system, gain-of-function mutations of *PTPN11* might play a role in the development of autoimmunity (200). Further to the above, SHP2 may mediate inhibitory receptor tyrosine kinase signaling in immune cells and the consequent inactivation of immunoregulatory receptors might contribute to the development of the autoimmune-like phenotypes (203), as well. Overall, activation of Ras signaling pathway in response to T cell receptor (TCR) stimulation is essential for T cell development, differentiation and activation. Proper regulation of Ras signal transduction plays a critical role in both normal immune responses and the maintenance of tolerance. On the other hand, both increased and decreased Ras activities may be implicated in lupus-like autoimmunity, while both under- and overactivities of disparate Ras effectors have been also linked to autoimmune responses (207) (Table 1).

NF1 is an autosomal dominant multisystemic disorder with an estimated incidence of ~1 in 2,500–3,000 individuals, affecting primarily the bone, the nervous system, soft tissue and the skin. About 50% of cases are caused by *de novo* (spontaneous) mutations. The diagnosis is usually made on clinical grounds, whereas genetic testing may be helpful in cases with unusual presentations or for reproductive decision-making. The presence of two or more of the following clinical features are required to establish a diagnosis of NF1: (1) ≥6 cafe'-au-lait macules >0.5 cm at largest diameter before puberty or >1.5 cm in diameter after puberty (2) axillary or inguinal freckling, (3) ≥2 Lisch nodules (4) ≥neurofibromas or ≥1 plexiform neurofibroma (5) an optic pathway glioma (OPG), (6) a distinctive osseous lesion (sphenoid wing dysplasia, long-bone dysplasia), and (7) a first-degree relative with NF1. NF1 patients have an increased risk of developing both benign and malignant tumors, including OPG, glioblastoma, malignant peripheral nerve sheath tumor, gastrointestinal stromal tumor, breast cancer, leukemia, phaeochromocytoma, duodenal carcinoid tumor, and rhabdomyosarcoma. Associated manifestations may include cardiovascular abnormalities, neurocognitive impairment and craniofacial dysmorphism (186, 208–210).

NF1 is caused by germline loss-of-function mutations in the *NF1* tumor-suppressor gene, located on chromosome 17q11.2 and encoding a large cytoplasmic protein called neurofibromin. Neurofibromin is a GTPase activating protein (GAP) that negatively regulates the Ras signal transduction pathway, by accelerating the conversion of active GTP-bound Ras to inactive GDP-bound Ras. Thus, loss of neurofibromin expression results in hyperactivation of RAS, as well as constitutive downstream MAPK and the mammalian target of rapamycin (mTOR) pathways (208, 211, 212).

Endocrinopathies are frequently observed in patients with NF1 and are usually related to OPGs involving the hypothalamic and sellar region and associated treatment modalities. Those may include short stature, central precocious puberty (CPP), diencephalic syndrome, GH and other pituitary deficiencies, GH hypersecretion and obesity with insulin resistance/impaired glucose tolerance (213, 214). Endocrine disorders were reported in 55.6% of children with NF1 and OPG who did not receive radiotherapy or surgical resection (214). Of note, hypopituitarism or CPP may occur even in the absence of intracranial lesions in NF1 patients. In this regard, GH deficiency was demonstrated in 15 out of 19 NF1 short children who had no intracranial tumors or other recognizable risk factors for short stature (215).

The number of studies reporting an association between NF1 and autoimmune disorders is small but increasing. NF1 cases with multiple sclerosis, SLE, membranous glomerulonephritis, IgA nephropathy, mixed connective tissue disease, myasthenia gravis, ankylosing spondylitis, JIA, CD, autoimmune hemolytic anemia, bullous pemphigoid, vitiligo, alopecia areata, T1D but also autoimmune thyroiditis and GD have been scarcely described in the literature (216–223) (Table 1). More recently, Güler et al., conducted a case-control study aiming to assess a possible relationship between NF1 and thyroid disorders. This study revealed that the frequency of autoimmune thyroiditis was not higher compared to the one reported in the general population. In particular, out of 78 NF1 children enrolled, 6.4% had goiter, 2.5% positive antithyroid antibodies, 1.2% SH and 3.8% low TSH levels, possibly related to impaired hypothalamo-hypophyseal-thyroid axis, which normalized after 1 year (224) (Table 1). With regards to the latter finding, TSH deficiency has been previously reported in NF1 children in the absence of intracranial tumors (225). It is worth mentioning, that neurofibromin functions as positive regulator of the enzyme adenylyl cyclase and cyclic adenosine monophosphate (cAMP) generation in the brain (213, 226). In particular, neurofibromin was shown to regulate hypothalamic function and pituitary development in the mammalian central nervous system by modulating intracellular cAMP levels. Interestingly, *NF1* inactivation in mice resulted in reduced body weights and anterior pituitary hypoplasia with a 40–60% reduction in the levels of growth hormone-releasing hormone (GHRH), gonadotropin-releasing hormone (GnRH) and TRH mRNA levels compared to wild type controls as well as reduced GH, prolactin and insulin-like growth factor 1 (IGF-1) mRNA levels (212).

The mechanisms underlying a possible association between autoimmune disorders and NF1 have not been elucidated. Studies in mice have shown that loss of neurofibromin results in decreased Fas antigen expression and Fas-ligand mediated apoptosis via hyperactivation of the p21ras-class IA PI-3K signaling pathway. Suppressed Fas ligand expression may prevent apoptosis of CD4+ T cells, which may contribute to the development of autoimmunity (219, 227). Furthermore, NF1-deficient mice exhibited lymphoproliferative defects, including thymic and splenic hyperplasia, increased numbers of immature and mature T cells *in vivo*, but reduced proliferation in response to TCR and interleukin-2 receptor (IL-2R) stimulation *in vitro* (228) as well as defective proliferative responses in B lymphocytes and thymocytes (229) (Table 1).

Overall, despite the lack of sufficient evidence, the growing number of reports on the concurrence of NF1 and autoimmune disorders implies a possible link between these conditions rather a coincidence.

Hypothyroidism/CH and thyroid hypoplasia have been scarcely described in the context of CFC, CS, CM-AVM, and Legius syndrome, however, to the best of our knowledge there are no reports on the coexistence of these RASopathies with ATD (230–233).

According to current recommendations for the care of children with NS, thyroid function tests including measurement of thyroid antibodies should be performed in all children with signs or symptoms of hypothyroidism (goiter, fatigue, constipation, poor growth, etc.) and every 3–5 years in older children and adults (194, 234). Thyroid disorders should be managed as in the general population (Table 2). Similarly, diagnosis and management guidelines for patients with CFC recommend thyroid function screening (TSH, FT4) at diagnosis, given that thyroid abnormalities can be observed in patients with other RASopathies and autoimmune thyroiditis is frequent in the general population. During follow-up thyroid function tests should be performed in case of growth failure. Only a few cases of hypothyroidism have been described in CFC, therefore the need for ongoing thyroid function testing can be evaluated by the endocrinologist (235) (Table 2). On the other hand, health supervision guidelines for NF1 children recommend annual evaluation of growth rate and pubertal development but lack information on thyroid function monitoring (236, 237). Taken into consideration (a) that TSH deficiency may be seen in affected individuals in the absence of intracranial tumors, (b) a possible although not well-documented association between NF1 and ATD, (c) the phenotypic similarities and common underlying pathogenetic etiology with NS and other RASopathies, it would be advisable to apply to NF1 patients the same recommendations on thyroid function monitoring as for NS patients (Table 2).

## Conclusion

In recent years, several research studies have shed light on the complex pathogenesis of genetic syndromes-associated ATD. The presence of chromosomal abnormalities may influence the phenotypic expression of thyroid autoimmunity, in particular with regards to age of onset of thyroidopathy, evolution of thyroid function and even clustering of extra-thyroidal autoimmune disorders in affected children. While thyroid dysfunction is namely of autoimmune origin in TS, DS, and KS, thyroid hypoplasia and central hypothyroidism account for the majority of cases in WS and PWS, respectively. Patients with 22q11.2DS have an increased risk for developing GD, however, thyroid gland anomalies are also frequently seen in affected individuals. In addition, there is emerging evidence supporting a possible link between autoimmunity and RASopathies. This comprehensive review highlights the need for an early screening, and regular life-long monitoring of the thyroid function in patients with specific genetic syndromes, along with a thorough evaluation that will enable early identification and proper management of both thyroid and extrathyroidal autoimmune disorders and associated endocrinopathies, in order to optimize the health care provided to these patients.

## Author Contributions

CK-G contributed to the study design, critically revised the manuscript, and approved the submitted version. EK was involved in the literature research and drafted the manuscript. All authors contributed to the article and approved the submitted version.

## Conflict of Interest

The authors declare that the research was conducted in the absence of any commercial or financial relationships that could be construed as a potential conflict of interest.
